# The Effect of Physical Exercise on Emotional Eating Among College Students: A Chain Mediation Analysis of Dietary Behavior and Body Satisfaction

**DOI:** 10.3390/bs16050727

**Published:** 2026-05-08

**Authors:** Qi-Yue Feng, Zi-Meng Guo, Hai-Ying Quan

**Affiliations:** 1 College of Physical Education, Liaoning Normal University, Dalian 116029, China; 15242632690@163.com (Q.-Y.F.); m18741253330_2@163.com (Z.-M.G.); 2 School of Physical Education, Guangzhou Sport University, Guangzhou 510500, China

**Keywords:** physical exercise, emotional eating, dietary behavior, body satisfaction, chain mediation

## Abstract

Objective: To investigate the relationship between physical exercise and emotional eating, as well as the chain mediating effects of dietary behavior and body satisfaction therein. Methods: A survey was conducted on 575 college students using the Physical Activity Level Scale, Dutch Eating Behavior Questionnaire, Body Self-State Scale, and Eating Behavior Scale. Results: (1) Physical exercise does not directly influence emotional eating. (2) Eating behavior mediated the relationship between physical exercise and emotional eating; body satisfaction did not mediate this relationship. (3) Both eating behavior and body satisfaction mediated the relationship between physical exercise and emotional eating. (4) Subgroup analysis revealed gender differences across all paths: mediating effects were significant among female participants but not among male participants. Conclusions: Findings reveal that physical exercise influences emotional eating through dietary behavior and the dietary behavior–body satisfaction pathway. This research provides insights for promoting college students’ physical and mental health and exploring the internal mechanisms of reducing emotional eating among this population.

## 1. Introduction

As social competition continues to intensify, college students face the dual pressures of academic demands and employment challenges, drawing increasing attention to their mental health. The prevalence of negative emotions such as anxiety and depression among college students is on the rise, reflecting the real challenges this group faces in psychological adaptation (Hua, 2020). In this context, eating disorders are becoming increasingly evident, emerging as one of the key factors threatening the physical and mental health of college students (Mushtaq et al., 2023). There exists a close connection between emotions and eating (Robbins & Fray, 1980), where stress can trigger eating. At the same time, it is important to note that not only negative emotions may lead to eating disorders, but positive emotions also carry the risk of causing abnormal dietary behavior (Bongers et al., 2013). Emotional eating, a coping mechanism that uses eating to alleviate emotional distress, is particularly prevalent among college students (Frayn & Knäuper, 2018). As a common manifestation of eating disorders, this behavior is typically accompanied by increased energy intake and heightened obesity risk. It readily triggers eating disorders in individuals and increases the likelihood of binge eating (Nunes et al., 2006). Research indicates that individuals who engage in emotional eating are more likely to develop eating disorders than those who do not (Evers et al., 2010). In this context, emotional eating refers to eating driven by emotions rather than hunger, using food to cope with negative feelings.

The National Health Plan for the 14th Five-Year Plan proposes that to advance public health, it is essential to effectively implement the National Nutrition Program and the Action for Balanced Diet, actively guide the public to enhance awareness of food appreciation, and foster balanced dietary habits across society (General Office of the State Council, 2022). Concurrently, efforts must be made to strengthen mental health and psychiatric service systems while reinforcing intervention mechanisms for psychological and behavioral issues. State Council General Office. Notice of the State Council General Office on Issuing the National Health Plan for the 14th Five-Year Plan. Against the backdrop of current policies, emotional eating—a common dietary disorder among college students closely linked to emotional states—holds significant practical value for exploring effective intervention strategies. Concurrently, the China Food and Nutrition Development Outline (2025–2030) highlights multiple challenges: insufficient supply and consumption of high-quality foods, residents yet to universally adopt scientific dietary habits, and simultaneous risks of both excessive and inadequate nutrient intake (Ministry of Agriculture and Rural Affairs of the People’s Republic of China, 2025). Against the backdrop of national policies emphasizing both “balanced diets” and “mental health,” coupled with the need to improve dietary habits among residents, guiding college students toward healthy dietary behavior has become a crucial entry point for implementing the national health strategy.

In this study, ‘dietary behavior’ refers to the relatively stable and observable eating patterns exhibited by individuals in everyday situations, driven by a combination of physiological needs and cognitive habits. Specifically, it encompasses dimensions such as the regularity of meals, the selection of food types, the quantity and frequency of intake, and eating styles. This dietary behavior reflects an individual’s routine, non-emotion-regulated eating habits in daily life, as distinct from eating activities undertaken to regulate or cope with emotional states. Emotional eating refers to eating behavior in which an individual is not driven by physiological hunger, but rather eats to regulate or cope with specific emotional states (particularly negative emotions such as stress, anxiety, depression and boredom). It constitutes a disordered eating pattern.

Within the conceptual framework of this study, emotional eating is not included as a sub-dimension of dietary behavior but is treated as an independent outcome variable. Dietary behavior is defined as routine eating habits and serves as a mediating variable between physical exercise and emotional eating, to examine whether physical exercise can reduce the occurrence of emotional eating by improving daily dietary behavior.

In short, emotional eating is a specific type of disordered eating behavior, whereas dietary behavior in this study measures routine daily eating patterns. Therefore, dietary behavior is used as a mediating variable between physical exercise and emotional eating to explore whether physical exercise can reduce the occurrence of emotional eating by improving routine dietary behavior.

In this study, dietary behavior refers to the observable and quantifiable patterns of conduct exhibited by individuals in daily life regarding food selection, acquisition, and consumption, shaped by the combined influence of physiological and cognitive drive systems (Rogers, 1999). Specifically, it encompasses the types, quantities, frequency, and methods of food consumption. The study distinguishes two driving dimensions of dietary behavior: routine dietary behavior, which is guided by physiological needs (homeostatic mechanisms), and cognitive control (planning and self-regulation), which is directed toward food itself.

The above conceptual definitions theoretically distinguish “eating for sustenance” from “eating for comfort” (emotional eating), providing a clear theoretical foundation for separately measuring dietary behavior and emotional eating in practice (Cannon & Washburn, 1912). This also suggests that optimizing dietary behavior not only improves nutritional health but also serves as an effective intervention for psychological issues like emotional eating.

Meanwhile, the National Health Plan for the 14th Five-Year Plan period also emphasizes the need to improve mental health services. An individual’s mental health is closely linked to their perception of their own image—that is, body satisfaction (Thompson & Schaefer, 2019). Among college students, body satisfaction not only influences mental health levels but also interacts profoundly with dietary behavior choices and patterns (Argyrides et al., 2025). In this research, body satisfaction serves as the core component of the affective dimension of body image. It encompasses both the subjective experience of one’s overall or specific body parts and the subjective evaluation of these perceptions. Its essence lies in the individual’s holistic perception of their body image and their overall level of satisfaction with it. Research data indicates that only 12.9% of college students in China express satisfaction with their body shape, while a significant 72.8% of female students express a desire to be thinner (Wang et al., 2018). A strong positive correlation exists between body satisfaction and unhealthy dietary behavior, with low body satisfaction often leading to a range of eating issues (Littleton & Ollendick, 2003; Murayama et al., 2021).

Physical exercise has been proven to be an effective means of emotional regulation, playing a positive role in reducing emotional eating tendencies (Luo, 2023). However, the relationship between the two is not a simple linear correspondence but may be influenced by multiple mediating or moderating factors, such as behavioral and psychological mechanisms including dietary behavior optimization, changes in emotional state, and improvements in body satisfaction. Therefore, this study aims to explore the specific pathways through which physical exercise influences emotional eating among college students, particularly examining the potential psychological and socio-cognitive mechanisms involved. The objective is to provide scientific evidence for alleviating emotional eating and promoting the development of healthy dietary behavior among college students.

## 2. Theoretical Hypotheses

### 2.1. The Relationship Between Physical Exercise and Emotional Eating

Emotion regulation theory posits that when individuals struggle to effectively manage their emotions, they may resort to overeating to temporarily alleviate negative emotions such as anxiety. This can gradually solidify emotional eating into a maladaptive coping mechanism, which is then reactivated in similar future situations (Evers et al., 2010). Meanwhile, the role of physical exercise in aiding emotion regulation appears to be a widely accepted fact. A significant positive correlation exists between physical exercise and emotion regulation capacity: higher levels of physical exercise correspond to stronger emotion regulation abilities in individuals. Research indicates that moderate-to-high intensity, longer duration, and higher frequency aerobic exercise, along with other types of physical activity, significantly impact anxiety reduction. As emotional eating serves as the primary coping strategy when emotional regulation fails, how physical exercise influences emotional eating becomes a critical research question. Based on this, the study proposes the following hypothesis:

**H1.**  *Physical exercise exerts a positive mitigating effect on emotional eating.*

### 2.2. The Relationship Between Physical Exercise, Dietary Behavior, and Emotional Eating

Extensive research indicates that poor dietary behaviors are inextricably linked to chronic diseases and significantly impact post-treatment recovery outcomes (Chai et al., 2019). A foreign study suggests that unhealthy eating habits can affect brain health and cognitive function; unbalanced dietary patterns negatively impact cognitive performance in healthy individuals during acute stress situations (Delgado et al., 2021). This leads individuals to confuse “physical hunger” with “emotional hunger” when anxious, thereby triggering emotional eating. Conversely, healthy eating patterns (such as the Mediterranean diet) are significantly associated with reduced anxiety and depression risk (Y. Li et al., 2017; Marchena et al., 2020; Moreno-Agostino et al., 2019). Balanced nutrition helps stabilize blood sugar levels, preventing emotional imbalances caused by glycemic fluctuations (Livesey et al., 2019). Mechanisms within the brain’s reward system indicate that early or prolonged high-fat diets can permanently alter its settings, leading individuals to frequently resort to emotional eating to cope with negative emotions (Teegarden et al., 2009). Long-term physical exercise can reduce appetite in obese individuals through various hormonal changes, thereby decreasing emotional eating (Xin et al., 2026). Based on this, the study proposes Hypothesis

**H2.**  *Dietary behavior mediates the relationship between physical exercise and emotional eating.*

### 2.3. The Relationship Between Physical Exercise, Body Satisfaction, and Emotional Eating

Body satisfaction, an affective dimension of body image, represents an individual’s comprehensive psychological response to their overall physique or specific body parts (Chen & Gao, 2005). This response encompasses both subjective perceptions (such as cognitive and emotional experiences) and subjective evaluations (such as attitudes and intentions). Essentially, it reflects an individual’s holistic subjective perception of their body image and their overall satisfaction with their physical appearance (Zhou, 2012). The sociocultural model of body satisfaction established by Thompson et al. (1999) reveals that an individual’s level of satisfaction with their physical appearance significantly influences the development of restrictive dietary behavior and binge eating symptoms. Research confirms that negative perceptions and evaluations of appearance among college students intensify the desire for thinness and the fear of weight gain, thereby triggering adverse dietary behavior such as restrictive dieting (Kong et al., 2013). Factors influencing body satisfaction can be categorized into three domains: individual psychological factors, individual physiological factors, and social environmental factors (Lee et al., 2025). Theoretically, physical exercise may affect body satisfaction through multiple pathways. Exercise can alleviate anxiety, enhance self-efficacy, and thereby improve an individual’s evaluation of their own body; regular exercise also helps control weight, improve posture, and enhance physical functions, and these objective changes directly impact the cognitive evaluation aspect of body satisfaction (Pan et al., 2026).

However, the relationship between physical exercise and body satisfaction does not hold across all dimensions. Some studies have subdivided female college students’ body self-satisfaction into five dimensions: athletic characteristics, appearance characteristics, body shape characteristics, sexual characteristics, and negative characteristics. The results (Sun, 2022) found that exercise volume was only significantly positively correlated with ‘athletic characteristics,’ while there was no significant correlation with appearance, body shape, or other dimensions. This suggests that the impact of physical exercise on body satisfaction may be mainly limited to the self-perception level directly related to athletic ability and may not directly enhance an individual’s satisfaction with appearance or overall body shape. Based on this, the study proposes hypothesis:

**H3.**  *Body satisfaction mediates the relationship between physical exercise and emotional eating.*

### 2.4. Chain-Mediated Hypothesis

In summary, physical exercise can alleviate anxiety and stabilize emotions by regulating hormones and related neurobiological mechanisms (Lin & Gao, 2023; Montgomery & Grant, 2026), thereby regulating an individual’s emotional state and ultimately exerting a positive effect on reducing emotional eating. According to Bandura’s self-efficacy theory, the “mastery experiences” accumulated through sustained participation in physical exercise enhance an individual’s “self-efficacy” in this domain—such as completing exercise plans or surpassing physical limits. This heightened sense of efficacy related to physical capability can further elevate overall body satisfaction. According to cognitive dissonance theory, when an individual’s actual behavior conflicts with their internal attitudes or self-concept, it creates an uncomfortable psychological tension (i.e., “cognitive dissonance”), motivating them to reduce this discomfort. For instance, cognitive dissonance may occur when an individual engages in physical exercise for health promotion but subsequently consumes large amounts of junk food. To alleviate this psychological discomfort, the individual may proactively optimize their dietary behavior. These theories collectively support the proposed chained mediating pathway: Physical Exercise → Dietary Behavior → Body Satisfaction → Emotional Eating.

Building upon this theoretical foundation, the study aims to investigate whether dietary behavior and body satisfaction mediate the relationship between physical exercise and emotional eating, and whether chain mediation exists between dietary behavior and body satisfaction. Research Hypothesis

**Figure 1:**
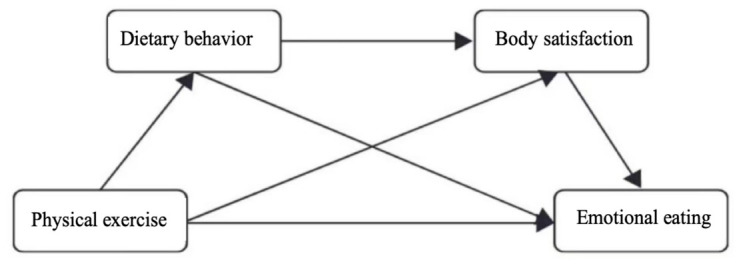
The Relationship Between Physical Exercise, Eating Behavior, Body Satisfaction, and Emotional Eating.

**H4.**  *A chain mediation pathway exists, wherein dietary behavior and body satisfaction act as chain mediators between physical exercise and emotional eating (Figure 1).*

## 3. Research Participants and Methods

### 3.1. Research Participants

Seventy invalid questionnaires were excluded based on the following criteria: (1) Logical inconsistencies between responses to forward and reverse-scored items in scales containing reverse-scored questions; (2) One or more scales exhibited consistent or identical response patterns. Ultimately, 575 valid questionnaires were recovered, yielding a response rate of 89.15%. Among these, 188 participants were male and 457 were female, with ages ranging from 18 to 32 and an average age of approximately 21.6. The demographic distribution of participants is presented in Table 1.

### 3.2. Research Tools

#### 3.2.1. Physical Activity Rating Scale (PARS-3)

Developed by Japanese scholar Hashimoto Kimio and revised by Chinese scholars including Liang (1994). This scale primarily assesses three dimensions of physical exercise: intensity, duration, and frequency, comprising a total of 5 items. Each item employs a 5-point Likert scale, with corresponding scores ranging from 1 to 5. The total physical exercise score is calculated as: Exercise Intensity Score × (Exercise Duration Score − 1) × Exercise Frequency Score, yielding a score range of 0 to 100 points. Higher scores indicate greater levels of physical exercise. In this study, the Cronbach’s alpha coefficient for this scale was 0.598.

#### 3.2.2. Dutch Eating Behavior Questionnaire (DEBQ)

The study employed the Chinese version of the DEBQ, revised by Y. Li et al. (2018) based on the original Dutch Eating Behavior Questionnaire developed by van Strien et al. (1986). This 33-item scale measures dietary behavior across three dimensions: restrictive eating (10 items), emotional eating (13 items), and external eating (10 items). The study measured the dimension of emotional eating (13 items), using a 5-point scoring method. A higher score indicates a stronger tendency toward emotional eating. This scale has demonstrated good reliability and validity in numerous domestic and international studies. In this study, the Cronbach’s alpha coefficient for the scale was 0.950.

#### 3.2.3. Body Image States Scale (BISS)

This scale was originally developed by Cash et al. (2002) to assess an individual’s body image state in specific situations. The Chinese version of the scale has undergone a standardized translation-back translation procedure by Cash et al. (2002). This questionnaire assesses satisfaction with body image states from six distinct perspectives using a 9-point Likert scale. Items 1, 3, and 5 employ a positive scoring method (1–9), while items 2, 4, and 6 use a reverse scoring method (9–1). Higher scores indicate greater satisfaction with body image states. According to R. L. Li (2022), a Cronbach’s α of 0.75 can be considered acceptable for research purposes, even though it is not high. Given that this scale has a small number of items and measures state body satisfaction, the Chinese version’s reliability coefficient of 0.75 falls within this acceptable range. In this study, the questionnaire demonstrated a Cronbach’s alpha coefficient of 0.826.

#### 3.2.4. Healthy Unhealthy Eating Behavior Scale (HUEBS)

Originally named the Healthy Unhealthy Eating Behavior Scale, this instrument was developed by Guertin et al. (2020) in Canada. It comprises 22 items, with 11 addressing healthy foods and the remaining 11 corresponding to foods recommended for moderate consumption in dietary guidelines. Participants are asked to select the frequency of consumption for each item based on their actual habits. Chinese scholar Di (2021) adapted and applied HUEBS to the Chinese context, translating and naming it the Dietary Behavior Scale. This provides additional assessment tools for domestic research on dietary behaviors. The study used this scale to measure dietary behavior, uses a 5-point Likert scale, requiring participants to rate each statement description based on their own situation from “1 Never” to “5 Always.” Higher scores indicate healthier dietary behaviors. In this study, the Cronbach’s alpha coefficient for the scale was 0.725.

### 3.3. Data Processing

Researchers first obtained informed consent from participants. After distributing and collecting questionnaires, participants were given small gifts. All questionnaires were collected immediately upon completion. Data processing and analysis were conducted using SPSS 31.0 statistical software with the PROCESS plugin. Primary statistical methods included descriptive statistics, difference tests, correlation analysis, regression analysis, and mediation effect testing. These methods aimed to systematically validate and interpret the intrinsic relationships among the four variables: physical exercise, emotional eating, dietary behavior, and body satisfaction.

## 4. Results

### 4.1. Descriptive Statistics

As shown in Table 2, males scored significantly higher in physical exercise (27.61 ± 21.65) than females (13.25 ± 15.04), t(572) = 9.05, *p* < 0.001; females scored significantly higher in emotional eating (33.71 ± 10.99) than males (30.43 ± 11.40), t(572) = −3.20, *p* = 0.001. There were no significant differences between genders in body satisfaction (*p* = 0.302) and eating behaviors (*p* = 0.562). The above results indicate significant gender differences in physical exercise and emotional eating, supporting the rationale for subsequent stratified analysis by gender.

### 4.2. Common Method Bias Test

The questionnaire data were derived from participants’ self-reports. To control for potential confounding effects of common method bias on the results, the study employed Harman’s single-factor test to examine this bias. Exploratory factor analysis was conducted on all items from the Physical Activity Level Scale, the Dutch Eating Behavior Questionnaire, the Body Self-State Scale, and the Eating Behavior Scale. Common factors were extracted using principal component analysis, and partial correlation coefficients were calculated by isolating the first common factor, as shown in Table 3. Results indicated only one factor with an eigenvalue greater than 1. The unrotated first factor explained 35.814% of variance, below the 40% threshold. Furthermore, after statistically controlling for the common method factor, partial correlation coefficients between all study variables remained significant (*p* < 0.001). The above analysis provides preliminary statistical support for common method bias, but given that all data in this study were obtained from self-reports and the study was cross-sectional in design, there may still be interference from common method bias, so the conclusions should be interpreted with caution.

### 4.3. Correlation Analysis of Physical Exercise, Eating Behavior, Self-Satisfaction, and Emotional Eating

Correlation analysis (Table 4) revealed significant correlations among all core variables. Emotional eating exhibited significant negative correlations with physical exercise (r = −0.113, *p* < 0.01), dietary behaviors (r = −0.196, *p* < 0.001), and body satisfaction (r = −0.149, *p* < 0.001). Simultaneously, the hypothesized chained mediating path was preliminarily validated: physical exercise was significantly positively correlated with dietary behavior (r = 0.169, *p* < 0.001), and dietary behavior were also significantly positively correlated with body satisfaction (r = 0.155, *p* < 0.001). Regarding control variables, gender showed a significant positive correlation with emotional eating (r = 0.132, *p* < 0.01) and a significant negative correlation with physical exercise (r = −0.354, *p* < 0.001). Grade level and only-child status did not reach statistical significance in their correlations with emotional eating.

### 4.4. Testing the Mediating Effects of Dietary Behavior and Body Satisfaction

Using stratified regression within multiple linear regression, we conducted regression analyses on physical exercise (independent variable), emotional eating (dependent variable), dietary behavior and body satisfaction (mediating variables), as well as gender, grade level, and only-child status (control variables). Chain mediation analysis was conducted using the SPSS PROCESS macro (Model 6), and the significance of indirect effects was tested using the bias-corrected percentile Bootstrap method (5000 resamples, 95% confidence interval).

In Model 1, gender (β = 0.117, *p* < 0.05) significantly positively influenced emotional eating, while grade level (β = −0.038, *p* < 0.05) and only child status (β = −0.055, *p* < 0.05) did not reach statistical significance. Additionally, physical exercise had no significant direct effect on emotional eating (β = −0.080, *p* < 0.05). In Model 2, after adding dietary behavior, the effect of gender remained consistent (β = 0.126, *p* < 0.01). Dietary behavior exerted a significant negative effect on emotional eating (β = −0.188, *p* < 0.001). In Model 3, with the addition of body satisfaction, both gender (β = 0.135, *p* < 0.01) and eating behavior (β = −0.170, *p* < 0.001) continued to show significant effects. Body satisfaction exerted a significant negative influence on emotional eating (β = −0.125, *p* < 0.01). The R^2^ values for Models 1 to 3 were 0.027, 0.061, and 0.076, respectively (Table 5).

**Figure 2:**
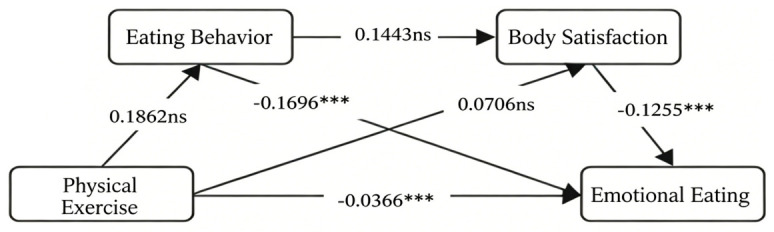
Chain Mediation Effect (*n* = 574). Note: ***: *p* < 0.001; ns: No significant effect.

Mediation results (Table 6) indicate that dietary behavior and body satisfaction exert indirect effects, with confidence intervals not containing zero. This confirms that both mediating variables significantly mediate the relationship between physical exercise and emotional problems. Multiple indirect effects constitute this mediation: The first indirect effect is Physical Exercise → Eating Behavior → Emotional Eating, with an effect value of −0.0193 and 95% CI = (−0.0353, −0.0065); The second indirect effect was physical exercise → body satisfaction → emotional eating, with an effect size of −0.0054 and 95% CI = (−0.0157, 0.0018). Since the confidence interval included zero, this path mediation effect was not significant. The third indirect effect was physical exercise → eating behavior → body satisfaction → emotional eating, with an effect value of −0.0021 and a 95% CI of (−0.0051, −0.0003), indicating a significant chain mediation effect. The contribution rates of the three paths were 39.39%, 11.02%, and 4.29%, respectively (Figure 2).

Analysis of the data in Table 4 reveals that gender is significant in all three models. Therefore, we conducted further analysis on the role of gender in the chained mediation. Using the SPSS PROCESS macro (Model 7), with gender as the moderator variable, the gender differences in indirect effects were tested by calculating the index of moderated mediation (IMM) and its 95% confidence interval (bias-corrected percentile bootstrap method, with 5000 resamples).

**Figure 3:**
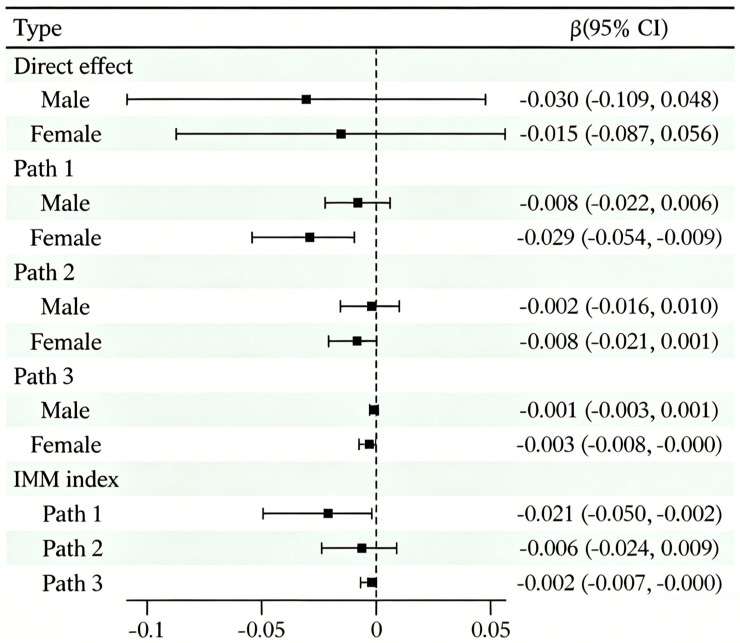
Subgroup Analysis Diagram (*n* = 574).

The results in Figure 3 clearly show that the direct effect of physical exercise on emotional eating was not significant for either males (β = −0.030, 95% CI [−0.109, 0.048]) or females (β = −0.015, 95% CI [−0.087, 0.056]); However, indirect path analysis revealed gender-specific mediating mechanisms: In Path 1, the path from physical exercise to emotional eating via dietary behavior was significant for females (β = −0.029, 95% CI [−0.054, −0.009]); In Path 3, the chain mediation from dietary behavior to body satisfaction was also significant (β = −0.003, 95% CI [−0.008, −0.000]). However, both paths were insignificant among males. Path 2 was insignificant for both males and females. The adjusted mediation indices (AMI) further confirmed the significance of Path 1 (IMM = −0.021, 95% CI [−0.050, −0.002]) and Path 3 (IMM = −0.002, 95% CI [−0.007, −0.000]) exhibited significant gender differences in indirect effects.

## 5. Discussion

Research findings indicate that physical exercise has no direct effect on emotional eating. However, the chained mediation model of physical exercise–dietary behavior–body satisfaction–emotional eating holds true, demonstrating that dietary behavior and body satisfaction fully mediate the relationship between physical exercise and emotional eating. When body satisfaction alone serves as the mediating variable between the two, the mediating effect is not significant.

### 5.1. Direct Effect of Physical Exercise on Emotional Eating

The study found an insignificant negative correlation between physical exercise and emotional eating, meaning that physical exercise cannot directly alleviate college students’ emotional eating behavior. The reason for this result may be that the effect of physical exercise on emotional eating is not primarily through a direct path, but mainly through indirect paths.

The chain mediation results of the study support this explanation. The results show that physical exercise has a significant indirect effect on emotional eating through dietary behavior (path 1) and the chain path of ‘dietary behavior → body satisfaction’ (path 3). When the mediator variables play a role in the model, the direct effect of physical exercise on emotional eating can be partially or entirely diverted. In this case, even if there is a real total effect between physical exercise and emotional eating, the direct path may no longer be significant due to the presence of the mediator variables.

Moreover, emotional eating itself involves using eating to regulate or escape unbearable negative emotions. If exercise also induces negative emotions in participants, it naturally intensifies this behavior. According to attentional replenishment theory, self-control resources inevitably deplete during exercise (D. Li & Zhang, 2020). Self-control represents an individual’s capacity to overcome impulses and desires to achieve long-term goals. After exercise, individuals’ psychological resources for self-regulation are depleted, reducing their capacity to manage negative emotions and increasing susceptibility to emotional eating. In summary, within college students’ daily lives, the level of exercise voluntariness, the intensity of physical activity, the abundance of individual self-control resources, and the strength of emotional regulation abilities all influence emotional eating behavior. Therefore, physical exercise is not directly related to emotional eating, but this does not mean that they are unrelated; rather, it indicates that the influence pathway is more complex—mainly exerting effects indirectly through mediating variables such as dietary behavior and body satisfaction. The chain mediation results of the study precisely support this view.

In summary, the effect of physical exercise on emotional eating is mainly realized indirectly by improving daily dietary behavior, thereby enhancing body satisfaction, rather than directly alleviating emotion-driven eating. This finding suggests that future intervention studies should not only focus on ‘increasing the amount of exercise,’ but should also simultaneously consider the synergistic effects of physical exercise and daily dietary behavior.

### 5.2. Mediating Effects of Eating Behavior

The core pathway through which physical exercise affects emotional eating is often realized by influencing an individual’s daily dietary behaviors rather than through a simple direct effect. Exercise may reshape an individual’s dietary behavior patterns in multiple ways. First, regular exercise can enhance an individual’s self-monitoring and executive functions, making them more sensitive to their food choices and thus more consciously avoiding high-sugar and high-fat foods. Studies show that acute moderate-to-high-intensity exercise can significantly improve inhibitory control, and this cognitive enhancement effect can transfer to the domain of dietary self-control—after exercise, individuals significantly reduce their intake of high-calorie snacks (Lowe et al., 2016). Secondly, physiological changes following exercise (such as regulation of gastrointestinal hormones and changes in appetite-related hormone levels) may naturally reduce cravings for energy-dense foods (Kirkpatrick et al., 2022). Systematic reviews indicate that long-term exercise tends to decrease rather than increase appetite, and this effect is particularly evident in people who exercise regularly (Konitz et al., 2024). In addition, longitudinal studies show that physical activity at the daily level is positively associated with healthy eating, meaning periods of higher physical activity are often accompanied by healthier food choices (Aulbach & Blechert, 2026). The concurrent occurrence of exercise and healthy eating on the same day supports the view that the two share self-regulatory resources. Finally, long-term adherence to physical exercise helps establish a sense of identification with a healthy lifestyle. The theory of cross-domain transfer of behavior change suggests that when an individual makes progress in one healthy behavior domain, it enhances their belief in their health capabilities, thereby motivating them to make efforts in another domain as well. To maintain the positive experiences brought by exercise, individuals often proactively adjust their dietary habits to form ‘exercise-health behavior’ consistency.

Therefore, physical exercise may first guide individuals toward healthier food preferences and selection habits (e.g., reducing high-fat/high-sugar foods, increasing nutrient-dense intake). Subsequently, this improved overall dietary pattern and quality can more effectively buffer negative emotions, ultimately intervening in emotional eating. This constitutes the core mediating pathway: physical exercise → dietary behavior → emotional eating. From the perspective of cognitive dissonance theory, this pathway also contains an intrinsic psychological driving force: when an individual defines themselves as a health-conscious person through physical exercise, unhealthy eating behaviors will conflict with this self-perception. To alleviate psychological discomfort, the individual will proactively adjust their eating behaviors to maintain self-consistency. In other words, the improvement of eating behaviors through physical exercise is based not only on physiological foundations but also involves cognitive self-regulation mechanisms.

### 5.3. Mediating Effect of Body Satisfaction

In the study, the independent mediating effect of body satisfaction between physical exercise and emotional eating was not significant, which is inconsistent with existing research. Further analysis showed that the reason for this non-significance is that the direct effect of physical exercise on body satisfaction is not significant. Although the direct effect of body satisfaction on emotional eating is significant, this also leads to the overall mediating path being non-significant. However, gender-specific analysis showed that the correlation between the two was significant among girls (r = 0.123, *p* = 0.013) but not significant among boys (r = 0.038, *p* = 0.628). This gender difference explains why the direct effect in the total sample was not significant—the lack of association between physical exercise and body satisfaction in the boys’ group lowered the overall effect size, resulting in the total sample correlation not reaching a significant level. This gender difference statistically explains why the direct effect in the total sample is not significant. From a mechanism perspective, the chain mediation model further reveals the pathway through which physical exercise affects body satisfaction.

The chain mediation pathway in the study (Path 3: Physical exercise → Eating behavior → Body satisfaction → Emotional eating) was found to be significant, indicating that the impact of physical exercise on body satisfaction is mainly achieved indirectly through improvements in daily eating behavior. In other words, when eating behavior is included as a mediator in the model, the direct effect of physical exercise on body satisfaction is no longer significant, and its impact is completely realized through the indirect pathway via eating behavior.

From a theoretical perspective, this finding aligns with the hierarchical logic of mediating variables. In the process of health behavior change, physical exercise typically first influences eating behaviors, which are directly related to the actions, and then affects more stable cognitive evaluations through improvements in body satisfaction. As an overall cognitive evaluation of body appearance and function, body satisfaction is usually formed and changed through the accumulation of specific behavioral changes rather than through the direct imparting of physical exercise. Moreover, the pattern where independent mediation effects are not significant but chain mediation effects are significant has important methodological implications. It suggests that when examining the mechanisms among multiple variables, a single mediation model may underestimate the actual contribution of some variables—when a variable is in a ‘downstream’ position in the causal chain, its independent mediation effect may not be significant, but as part of a chain pathway, it can play a key role. Therefore, the non-significance of Path 2 does not imply that body satisfaction is unimportant; rather, it indicates that in the relationship between physical exercise and emotional eating, body satisfaction needs to work in conjunction with eating behavior.

In summary, the independent mediation effect of body satisfaction is not significant, which precisely verifies the theoretical assumption of this study: the influence of physical exercise on emotional eating is a chain process rather than a single mediation path. As the latter part of this chain, the effect of body satisfaction relies on prior changes in eating behavior. This finding provides a more refined theoretical framework for understanding the multiple pathways through which physical exercise affects emotional eating.

### 5.4. Chain Mediation Effect of Eating Behavior–Body Satisfaction

The chain mediation model of physical exercise–dietary behavior–body satisfaction–emotional eating reveals the pivotal role of dietary behavior–body satisfaction in influencing emotional eating among female college students during daily exercise. Specifically, dietary behavior, as a mediating variable that channels the positive effects of physical exercise, enhances body satisfaction among female college students, thereby laying the groundwork for alleviating emotional eating.

The role of physical exercise in alleviating emotional eating manifests through two synergistic pathways: psychological and physiological. From the psychological perspective, regular exercise significantly enhances individuals’ self-efficacy and emotional regulation abilities. According to Bandura’s self-efficacy theory, this sense of efficacy mainly comes from ‘mastery experiences’ in exercise, and it can generalize from the exercise domain to the dietary behavior domain—when individuals believe they are capable of maintaining exercise, they are also more confident in improving their eating habits; and the improvement in physical condition brought by healthy eating further strengthens body satisfaction. These positive psychological pathways encourage individuals to proactively choose healthier diets in daily life, thereby gradually increasing their body satisfaction and reducing tendencies toward emotional eating. From the physiological pathway, physical exercise promotes the spontaneous formation of healthy dietary behavior by regulating appetite-related hormones and food reward mechanisms (Stensel, 2011; Beaulieu et al., 2020; Bilski et al., 2009). Subsequently, the stable physiological state supported by healthy eating—such as steady blood sugar levels and reduced inflammation—directly improves an individual’s body satisfaction. This, in turn, alleviates emotional eating through enhanced emotional regulation (Ripoli et al., 2022; Kong et al., 2013).

In summary, these two pathways are not isolated but mutually reinforcing and synergistic. Enhanced psychological self-control facilitates adherence to healthy eating, while physiological appetite normalization and improved physical condition, in turn, consolidate positive body experiences and emotional regulation capabilities. This integrated mechanism not only provides female college students with multiple pathways and tools to alleviate emotional eating but also offers robust theoretical support for cultivating better exercise habits and enhancing self-efficacy by fostering healthier physical and psychological states. It further provides new perspectives and insights for exploring the internal mechanisms through which physical exercise influences emotional eating.

## 6. Conclusions and Recommendations

### 6.1. Conclusions

The study reveals a chain mediation effect between dietary behavior and body satisfaction in the relationship between physical exercise and emotional eating. Physical exercise does not directly influence emotional eating but must rely on the chain mediation effects of dietary behavior and body satisfaction. Subgroup analysis indicates gender differences in this chain mediation model: the chain mediation effect holds for female students, with the effect mediated by dietary behavior being particularly significant. In males, the mediating effects were not established. Therefore, when addressing emotional eating among female college students, interventions should focus on dietary behaviors. This includes enhancing awareness of healthy eating, disseminating scientific knowledge about nutrition, and encouraging campus cafeterias to provide more scientifically balanced meals. These measures can help students improve their dietary habits, ultimately reducing emotional eating. It should be noted that the reliability of the physical exercise scale in this study (α = 0.598) is relatively low, and measurement error usually causes effect estimates to be biased toward conservatism. Therefore, the above conclusions should be interpreted within this limitation, and more precise measurement tools need to be used for verification in the future.

### 6.2. Recommendations

The questionnaire-based measurement in this study relies on self-reporting, potentially introducing recall bias. The proportion of female participants in the study was relatively high, which may have some impact on the stability of the results of gender difference analysis. Future research could further balance the gender sample to enhance the robustness of the conclusions. In addition, the reliability of the physical activity scale was relatively low, which may affect the robustness of the results. It is recommended that future research uses more precise measurement tools for verification. Constrained by its cross-sectional design, the research faces limitations in rigorously inferring causal relationships between variables, thereby restricting a thorough explanation of the mechanisms through which physical exercise influences emotional eating. Future research is recommended to employ longitudinal tracking or experimental designs to further validate the reliability and causal direction of the findings. In terms of mediating mechanisms, research mainly focuses on the mediating role of dietary behavior and body satisfaction, with limited explanatory power for emotional eating. Future studies may consider including more relevant psychological and behavioral variables to more comprehensively reveal the mechanism between physical exercise and emotional eating. Subsequent studies should consider incorporating additional relevant psychological and behavioral variables to more comprehensively elucidate the mechanisms linking physical exercise and emotional eating. Research indicates that the direct effects of physical exercise on emotional eating and body satisfaction have not been consistently confirmed across all studies. This seemingly contradictory finding suggests that the underlying mechanisms may be influenced by a complex interplay of mediating and moderating variables, rather than simple direct causal relationships. Specifically, the degree of voluntary exercise and self-control resources may play crucial roles in mediating the relationship between physical exercise and emotional eating. In the relationship between physical exercise and body satisfaction, the nature of exercise motivation and changes in dietary behavior may constitute important pathways. Additionally, dividing body satisfaction into distinct dimensions may facilitate identifying the internal mechanisms through which physical exercise influences body satisfaction. Therefore, future research should integrate multiple variables—including exercise motivation, voluntary engagement levels, self-control resources, and dietary behaviors—while conducting dimensional analyses of body satisfaction. By constructing comprehensive path models, we can systematically elucidate how physical exercise ultimately influences emotional eating through multiple psychological and behavioral mediating processes. This approach will provide robust theoretical and empirical foundations for designing and implementing targeted, integrated mind–body intervention programs.

## Figures and Tables

**Table 1:** Basic Information (*n* = 574).

Demographic Variables	Class	Number of People	Percentage
Grade	Freshman	125	19.38%
	Sophomore	116	17.98%
	Junior	52	8.06%
	Senior	51	7.91%
	First Year Of Graduate School	165	25.58%
	Second Year Of Graduate School	80	12.40%
	Third Year Of Graduate School	46	7.13%
	Doctor	10	1.55%
The Only Child	The Only Child	315	48.84%
	Non-Only Child	330	51.16%

**Table 2:** Descriptive statistics of each core variable grouped by gender (M ± SD) (*n* = 574).

Gender	Physical Exercise	Dietary Behavior	Body Satisfaction	Emotional Eating
Male	27.61 ± 21.65	70.87 ± 8.31	32.60 ± 7.79	30.43 ± 11.40
Female	13.25 ± 15.04	70.39 ± 9.15	33.29 ± 7.03	33.71 ± 10.99
Total Sample	17.35 ± 18.36	70.52 ± 8.91	33.09 ± 7.25	32.78 ± 11.20

**Table 3:** Determine partial correlation results by separating the first common factor (*n* = 574).

		Physical Exercise	Dietary Behavior	Body Satisfaction	Emotional Eating
Physical Exercise	relativity	1	−0.298	−0.298	0.324
Dietary Behavior	relativity	−0.298	1	−0.354	0.419
Body Satisfaction	relativity	−0.298	−0.354	1	0.301
Emotional Eating	relativity	0.324	0.419	0.301	1

**Table 4:** Correlation between Variables (*n* = 574).

Variable	Sex	Grade	The Only Child	Physical Exercise	Dietary Behavior	Body Satisfaction	Emotional Eating
Sex	1						
Grade	0.216 ***	1					
The Only Child	0.084 *	−0.029	1				
Physical Exercise	−0.354 ***	−0.029	−0.130 **	1			
Dietary Behavior	−0.024	−0.033	−0.009	0.169 ***	1		
Body Satisfaction	0.043	−0.002	0.007	0.068	0.155 ***	1	
Emotional Eating	0.132 **	−0.009	−0.034	−0.113 **	−0.196 ***	−0.149 ***	1

Note: * *p* < 0.05; ** *p* < 0.01; *** *p* < 0.001. The same applies below.

**Table 5:** Regression Results of Chain Mediation Effect (*n* = 574).

Variable	Model 1			Model 2			Model 3		
	b	SE	β	b	SE	β	b	SE	β
Sex	2.894	1.124	0.117 *	3.122	1.106	0.126 **	3.349	1.101	0.135 **
Grade	−0.209	0.233	−0.038	−0.248	0.229	−0.045	−0.255	0.228	−0.047
Only Son	−1.241	0.935	−0.055	−1.198	0.92	−0.054	−1.168	0.913	−0.052
Physical Exercise	−0.049	0.027	−0.08	−0.028	0.027	−0.045	−0.022	0.027	−0.037
Dietary Behavior				−0.236	0.052	−0.188 ***	−0.213	0.052	−0.170 ***
Body Satisfaction							−0.194	0.063	−0.125 **
R^2^	0.027			0.061			0.076		

Note: * *p* < 0.05; ** *p* < 0.01; *** *p* < 0.001. The same applies below.

**Table 6:** Direct and Indirect Paths to Standardization (*n* = 574).

Effect And Path	Effect Size	Boot Standard Error	Bootstrap 95% CI		Relative Mediating Effect
			Lower Limit	Superior Limit	
Gross Effect	−0.0490	0.0272	−0.1024	0.0043	100.00%
Direct Effect	−0.0223	0.0270	−0.0753	0.0307	45.51%
Aggregate Intermediary Effect	−0.0267	0.0092	−0.0463	−0.0104	54.49%
Physical Exercise–Dietary Behavior–Emotional Eating	−0.0193	0.0074	−0.0353	−0.0065	39.39%
Physical Exercise–Body Satisfaction–Emotional Eating	−0.0054	0.0044	−0.0157	0.0018	11.02%
Physical Exercise–Dietary Behavior–Body Satisfaction–Emotional Eating	−0.0021	0.0013	−0.0051	−0.0003	4.29%

Note: 5000 Bootstrap samples.

## Data Availability

All data supporting this study are included in the article.

## References

[B1-behavsci-16-00727] Argyrides M., Efthyvoulou L., Zamba K., Anastasiades E., Charalambous Z. (2025). Influences of sex and BMI on body image, weight bias, disordered eating, and psychological well-being: A multivariate analysis. Obesities.

[B2-behavsci-16-00727] Aulbach M. B., Blechert J. (2026). Positive associations between day-level physical activity and healthy eating in participants of a health promotion course: An exploratory study. Journal of Behavioral Medicine.

[B3-behavsci-16-00727] Beaulieu K., Oustric P., Finlayson G. (2020). The impact of physical activity on food reward: Review and conceptual synthesis of evidence from observational, acute, and chronic exercise training studies. Current Obesity Reports.

[B4-behavsci-16-00727] Bilski J., Teległów A., Zahradnik-Bilska J., Dembiński A., Warzecha Z. (2009). Effects of exercise on appetite and food intake regulation. Medicina Sportiva.

[B5-behavsci-16-00727] Bongers P., Jansen A., Havermans R., Roefs A., Nederkoorn C. (2013). Happy eating. The underestimated role of overeating in a positive mood. Appetite.

[B6-behavsci-16-00727] Cannon W. B., Washburn A. L. (1912). An explanation of hunger. American Journal of Physiology-Legacy Content.

[B7-behavsci-16-00727] Cash T. F., Fleming E. C., Alindogan J., Steadman L., Whitehead A. (2002). Beyond body image as a trait: The development and validation of the body image states scale. Eating Disorders.

[B8-behavsci-16-00727] Chai Y., Yang H., Wu T., Zhang L., Liang F., Li M. (2019). Nursing outcomes of discharged patients with chronic obstructive pulmonary disease and the associated factors. Journal of Nursing Science.

[B9-behavsci-16-00727] Chen H., Gao X. (2005). Body image disorder and influencing factors among college students. Health Medicine Research and Practice.

[B10-behavsci-16-00727] Delgado I., Dexpert S., Sauvant J., Cryan J. F., Capuron L. (2021). Influence of pro-obesogenic dietary habits on stress-induced cognitive alterations in healthy adult volunteers. Neurobiology of Stress.

[B11-behavsci-16-00727] Di L. (2021). Localization and application of the Chinese version of the dietary motivation questionnaire. Doctoral thesis.

[B12-behavsci-16-00727] Evers C., Marijn Stok F., de Ridder D. T. D. (2010). Feeding your feelings: Emotion regulation strategies and emotional eating. Personality and Social Psychology Bulletin.

[B13-behavsci-16-00727] Frayn M., Knäuper B. (2018). Emotional eating and weight in adults: A review. Current Psychology.

[B14-behavsci-16-00727] General Office of the State Council (2022). Notice of the general office of the state council on issuing the 14th five-year plan for national health.

[B15-behavsci-16-00727] Guertin C., Pelletier L., Pope P. (2020). The validation of the Healthy and Unhealthy Eating Behavior Scale (HUEBS): Examining the interplay between stages of change and motivation and their association with healthy and unhealthy eating behaviors and physical health. Appetite.

[B16-behavsci-16-00727] Hua W. (2020). A study on the current status of depression, anxiety, and stress among college students. Doctoral thesis.

[B17-behavsci-16-00727] Kirkpatrick G. E., Dingess P. M., Aadland J. A., Brown T. E. (2022). Acute high-intensity interval exercise attenuates incubation of craving for foods high in fat. Obesity.

[B18-behavsci-16-00727] Kong F., Zhang Y., You Z., Fan C., Tian Y., Zhou Z. (2013). Body dissatisfaction and restrained eating: Mediating effects of self-esteem. Social Behavior and Personality: An International Journal.

[B19-behavsci-16-00727] Konitz C., Schwensfeier L., Predel H. G., Brinkmann C. (2024). The influence of acute and chronic exercise on appetite and appetite regulation in patients with prediabetes or type 2 diabetes mellitus—A systematic review. Nutrients.

[B20-behavsci-16-00727] Lee M. F., Bolton K., Madsen J., Burke K. J. (2025). A systematic review of influences and outcomes of body image in postpartum via a socioecological framework. Journal of Reproductive and Infant Psychology.

[B21-behavsci-16-00727] Li D., Zhang L. (2020). Inhibition and persistence self-control in cognitive and motor tasks under improved natural environment. China Sports Science and Technology.

[B22-behavsci-16-00727] Li R. L. (2022). She hui gui fan dui nü xing shen ti man yi du de ying xiang *[The influence of social norms on women’s body satisfaction]*. Master’s thesis.

[B23-behavsci-16-00727] Li Y., Liu Y., Bao J. (2018). The applicability of the Chinese version of the Dutch Eating Behavior Questionnaire in the population of Chinese college students. Chinese Journal of Clinical Psychology.

[B24-behavsci-16-00727] Li Y., Lv M. R., Wei Y. J., Sun L., Zhang J. X., Zhang H. G., Li B. (2017). Dietary patterns and depression risk: A meta-analysis. Psychiatry Research.

[B25-behavsci-16-00727] Liang D. (1994). The stress level of college students and its relationship with physical exercise. Chinese Mental Health Journal.

[B26-behavsci-16-00727] Lin Y., Gao W. (2023). The effects of physical exercise on anxiety symptoms of college students: A meta-analysis. Frontiers in Psychology.

[B27-behavsci-16-00727] Littleton H. L., Ollendick T. (2003). Negative body image and disordered eating behavior in children and adolescents: What places youth at risk and how can these problems be prevented?. Clinical Child and Family Psychology Review.

[B28-behavsci-16-00727] Livesey G., Taylor R., Livesey H. F., Buyken A. E., Jenkins D. J. A., Augustin L. S. A., Sievenpiper J. L., Barclay A. W., Liu S., Wolever T. M. S., Willett W. C., Brighenti F., Salas-Salvadó J., Björck I., Rizkalla S. W., Riccardi G., Vecchia C. L., Ceriello A., Trichopoulou A., Brand-Miller J. C. (2019). Dietary glycemic index and load and the risk of type 2 diabetes: A systematic review and updated meta-analyses of prospective cohort studies. Nutrients.

[B29-behavsci-16-00727] Lowe C. J., Kolev D., Hall P. A. (2016). An exploration of exercise-induced cognitive enhancement and transfer effects to dietary self-control. Brain and Cognition.

[B30-behavsci-16-00727] Luo S. (2023). The relationship between physical exercise and college students’ subjective well-being: The mediating effect of emotion regulation self-efficacy. Sports World.

[B31-behavsci-16-00727] Marchena C., Bernabéu E., Iglesias M. T. (2020). Are adherence to the Mediterranean diet, emotional eating, alcohol intake, and anxiety related in university students in Spain?. Nutrients.

[B32-behavsci-16-00727] Ministry of Agriculture and Rural Affairs of the People’s Republic of China (2025). Notice of the ministry of agriculture and rural affairs, national health commission, and ministry of industry and information technology on issuing the China food and nutrition development outline (2025–2030)..

[B33-behavsci-16-00727] Montgomery T. R., Grant D. M. (2026). Neurobiological, molecular, and systemic mechanisms of exercise in the treatment of mental health disorders. Journal of Psychiatric Research.

[B34-behavsci-16-00727] Moreno-Agostino D., Caballero F. F., Martín-María N., Tyrovolas S., López-García P., Rodríguez-Artalejo F., Haro J. M., Ayuso-Mateos J. L., Miret M. (2019). Mediterranean diet and wellbeing: Evidence from a nationwide survey. Psychology & Health.

[B35-behavsci-16-00727] Murayama Y., Ito H., Hamada M., Takayanagi N., Myogan M., Suzuki K., Tsujii M. (2021). Examining simultaneous associations of four emotion regulation strategies with abnormal eating behaviors/attitudes in early adolescents. Eating Behaviors.

[B36-behavsci-16-00727] Mushtaq T., Ashraf S., Hameed H., Irfan A., Shahid M., Kanwal R., Aslam M. A., Shahid H., Koh-E-Noor, Shazly G. A., Khan M. A., Jardan Y. A. B. (2023). Prevalence of eating disorders and their association with social media addiction among youths. Nutrients.

[B37-behavsci-16-00727] Nunes M. A., Olinto M. T. A., Camey S., Morgan C., de Jesus Mari J. (2006). Abnormal eating behaviors in adolescent and young adult women from southern Brazil: Reassessment after four years. Social Psychiatry and Psychiatric Epidemiology.

[B38-behavsci-16-00727] Pan X., Jiang L., Zhang Y., Suzuki K., Gao Y., He J., Chen X., Zhang A. (2026). The impact of exercise self-efficacy, self-esteem and physical activity on body fat percentage changes in adolescents during fat loss interventions. Scientific Reports.

[B39-behavsci-16-00727] Ripoli C., Ricciardi M. R., Zuncheddu E., Angelo M. R., Pinna A. P., Ripoli D. (2022). Emotional eating and disordered eating behaviors in children and adolescents with type 1 diabetes. Scientific Reports.

[B40-behavsci-16-00727] Robbins T. W., Fray P. J. (1980). Stress-induced eating: Fact, fiction or misunderstanding?. Appetite.

[B41-behavsci-16-00727] Rogers P. J. (1999). Eating habits and appetite control: A psychobiological perspective. Proceedings of the Nutrition Society.

[B42-behavsci-16-00727] Stensel D. (2011). Exercise, appetite and appetite-regulating hormones: Implications for food intake and weight control. Annals of Nutrition and Metabolism.

[B43-behavsci-16-00727] Sun H. (2022). A study on the impact of physical exercise on female college students’ body self-satisfaction and general self-efficacy. Master’s thesis.

[B44-behavsci-16-00727] Teegarden S. L., Scott A. N., Bale T. L. (2009). Early life exposure to a high fat diet promotes long-term changes in dietary preferences and central reward signaling. Neuroscience.

[B45-behavsci-16-00727] Thompson J. K., Heinberg L. J., Altabe M., Tantleff-Dunn S. (1999). Exacting beauty: Theory, assessment, and treatment of body image disturbance.

[B46-behavsci-16-00727] Thompson J. K., Schaefer L. M. (2019). Thomas F. Cash: A multidimensional innovator in the measurement of body image; Some lessons learned and some lessons for the future of the field. Body Image.

[B47-behavsci-16-00727] van Strien T., Frijters J. E. R., Bergers G. P. A., Defares P. B. (1986). The Dutch Eating Behavior Questionnaire (DEBQ) for assessment of restrained, emotional, and external eating behavior. International Journal of Eating Disorders.

[B48-behavsci-16-00727] Wang K., Liang R., Ma Z. L., Chen J., Cheung E. F. C., Roalf D. R., Gur R. C., Chan R. C. K. (2018). Body image attitude among Chinese college students. PsyCh Journal.

[B49-behavsci-16-00727] Xin X., Liu L., Guo Y., Wang H., Xie J. (2026). Effects of long-term exercise interventions on appetite-regulating hormones in overweight and obese populations: A meta-analysis. Chinese Journal of Tissue Engineering Research.

[B50-behavsci-16-00727] Zhou Y. (2012). The impact of idealized thinness internalization on body satisfaction in young women. Doctoral thesis.

